# The Great Mimicker, a Rare Pleural Presentation

**DOI:** 10.1016/j.chpulm.2025.100176

**Published:** 2025-07-25

**Authors:** Jack McCarthy, Niamh Boyle, Mark Coyne, Cormac McCarthy

**Affiliations:** a Department of Respiratory Medicine, St. Vincent’s University Hospital, Dublin, Ireland; b Department of Haematology, St. Vincent’s University Hospital, Dublin, Ireland; c School of Medicine, University College Dublin, Dublin, Ireland

## Abstract

A 75-year-old woman presented to the emergency department of a regional hospital with progressive dyspnea for 3 months despite treatment with antibiotics and steroids in the community. There were no associated symptoms. No clear precipitating factors were identified. She did not smoke and had worked as an arable farmer but had no known exposures. Her medical history was remarkable for hypertension and a prophylactic hysterectomy 10 years previously due to a family history of ovarian cancer. Her medications included olmesartan, lansoprazole, and calcium supplements.

## Physical Examination Findings

Initial vital signs were temperature of 98 °F, BP of 125/55 mm Hg, heart rate of 85 beats/min, respiratory rate of 22 breaths/min, and oxygen saturation of 97% on room air. Chest examination revealed reduced air entry on the right side to the midzone with vesicular breathing on the left. There were normal heart sounds with no appreciable murmurs, no raised jugular venous pulse, and no peripheral edema. Abdominal examination was unremarkable with no palpable organomegaly. Of note, there was no clubbing, synovitis, or rashes visible, but there was palpable right axillary and inguinal adenopathy.

## Diagnostic Studies

**Figure 1:**
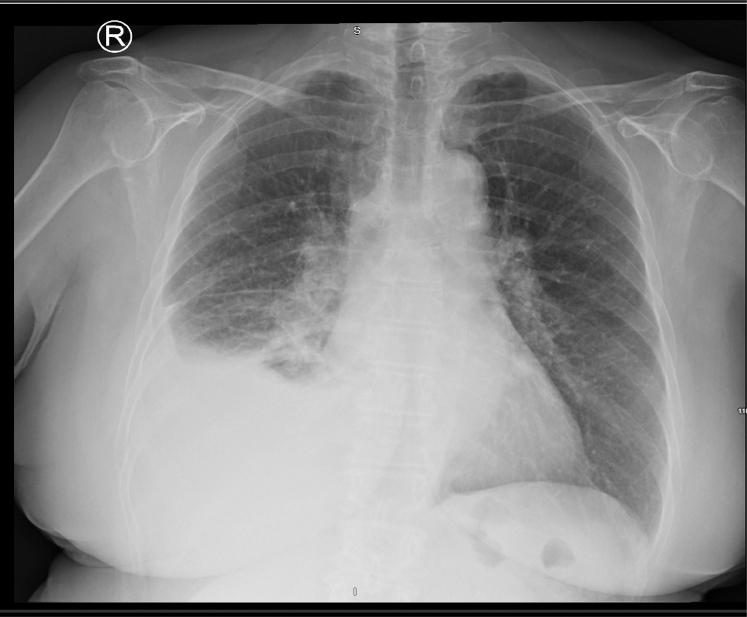
Posteroanterior(PA) chest radiograph showing moderate to large volume pleural effusion on the right side with some overlying consolidation.

Initial investigations included hemoglobin 14.4 g/dL, platelets 278 × 10^9^/L, and WBC count 5.5 × 10^9^/L with normal differential. Her biochemistry profile and C-reactive protein were also in the normal range. A chest radiograph demonstrated a large right-sided pleural effusion (Fig 1). An echocardiogram demonstrated preserved left ventricular function without valvular abnormalities. Ultrasound-guided thoracocentesis demonstrated a lymphocytic predominant exudative effusion. Initial microbiology did not yield any pathogens, and initial cytology was negative for malignancy. An ultrasound-guided pleural drain was inserted due to worsening dyspnea.

**Figure 2:**
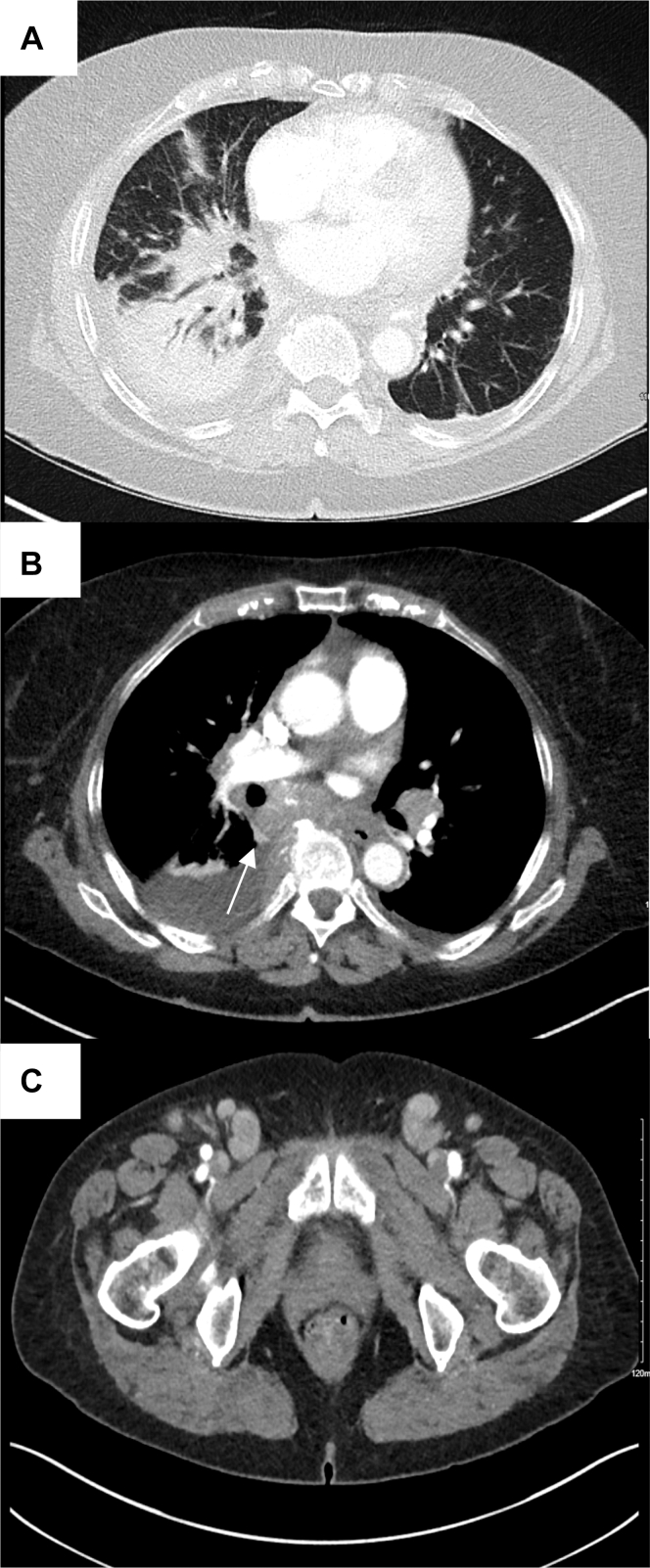
A, Axial CT thorax, abdomen, and pelvis lung windows showing a large partially loculated pleural effusion with extensive pleural nodularity and interlobular septal thickening along with diffuse pulmonary nodularity. B, Axial soft tissue windows from the same CT scan showing infiltrating mass around the right hilum with partially calcified hilar, subcarinal, and paraoesophageal lymph nodes (white arrow). C, Axial soft tissue window from the same CT scan showing bulky inguinal lymphadenopathy with the largest on the left 2.8 cm.

CT scan of the thorax, abdomen, and pelvis was performed, which showed a loculated pleural effusion (Figs 2A-C) along with some partially calcified mediastinal lymphadenopathy and inguinal lymphadenopathy. The chest drain remained in situ for 5 days with good symptomatic relief after drainage. An excisional biopsy was then performed of the lymph nodes, and the patient was discharged to await the histopathology.

She presented again 4 weeks later with worsening dyspnea and recurrence of the large right-sided effusion requiring repeat thoracocentesis. Pleural fluid analysis again showed no evidence of infection, TB, carcinoma, or lymphoma. Lymph node histopathology showed amyloid deposits in keeping with amyloidosis.

Further investigations included serum protein electrophoresis demonstrating 2 paraprotein bands detected in gamma globulin fraction and 1 in the beta globulin fraction, and an elevated IgM of 22.29 g/L. Hemoglobin, C-reactive protein, calcium, and lactate dehydrogenase(LDH) were within normal limits.

The patient was referred to a tertiary referral center for multidisciplinary discussion. The outcome was that further investigation was warranted to differentiate between amyloid lymphadenopathy due to primary systemic amyloidosis or reactive systemic amyloidosis possibly secondary to lymphoma, myeloma, or carcinoma. Due to this uncertainty, CT scans for skeletal survey and myeloma were undertaken, which were not suggestive of multiple myeloma. A video assisted thorascopic surgery(VATS) pleurodesis was performed with pleural biopsy done at the same time. This demonstrated inflamed parietal pleura showing diffuse amyloid deposits. Congo red staining was positive with apple-green birefringence on polarized light for amyloid; however, the question remained whether this was due to primary or reactive systemic amyloidosis.

*What is the diagnosis?*

*Diagnosis:* Systemic amyloid light chain (AL ) amyloidosis

## Discussion

This case reports an unusual case of systemic AL amyloidosis presenting with a unilateral pleural effusion. This is interesting because systemic amyloidosis is a rare condition. Recurrent pleural effusion without cardiomyopathy as a manifestation of amyloidosis is even rarer, occurring in 1% to 2% of cases.

Pleural effusions associated with amyloidosis are often refractory to diuresis and drainage. The mechanism of amyloidosis causing pleural effusion is not clearly established. Effusions secondary to amyloidosis can be transudates or exudates and can be unilateral or bilateral. If the pleural effusion is a transudate, the mechanism of the effusion can be secondary to systemic factors (eg, congestive heart failure due to cardiac amyloid involvement, nephrotic syndrome due to renal amyloid involvement, hypoalbuminemia due to liver involvement). It can also be due to amyloid material blocking lymphatic flow and the deposition of amyloid material in the pulmonary blood vessels leading to increased pulmonary venous pressure. If the pleural effusion is exudate, amyloid infiltration of the pleural cavity causes diffuse pleural inflammation, which causes leakage of fluid into the pleural space and impairs drainage from the pleural cavity.

Amyloidosis is caused by misfolding of autologous proteins, which are deposited extracellularly as fibrils resulting in organ dysfunction. Clinical manifestations of different forms of amyloidosis can be similar but their treatment varies, and this is based on the amyloidogenic precursor or pathogenic mechanism. Therefore, definitive identification of the subset of amyloid is crucial to avoid therapeutic errors.

Diagnosis of amyloidosis should be based on tissue biopsy and multidisciplinary team discussion. As shown in this case, the lymph node, endobronchial, and bone marrow biopsies all showed evidence of amyloidosis and were discussed at the multidisciplinary team discussion. The affected tissue typically shows apple-green birefringence after Congo red staining under polarized light, which is the criterion standard for diagnosing amyloidosis.

The possibility of amyloidosis should be considered when a refractory pleural effusion or undetermined effusion is encountered, and consideration should be given to thoracoscopic examination. There are little robust data to support a specific approach to management of refractory pleural effusion in systemic amyloidosis; however, a combination of systemic chemotherapy along with use of talc pleurodesis or indwelling pleural catheter (IPC) may be the best choice of management given current data.

### Clinical Course

**Figure 3:**
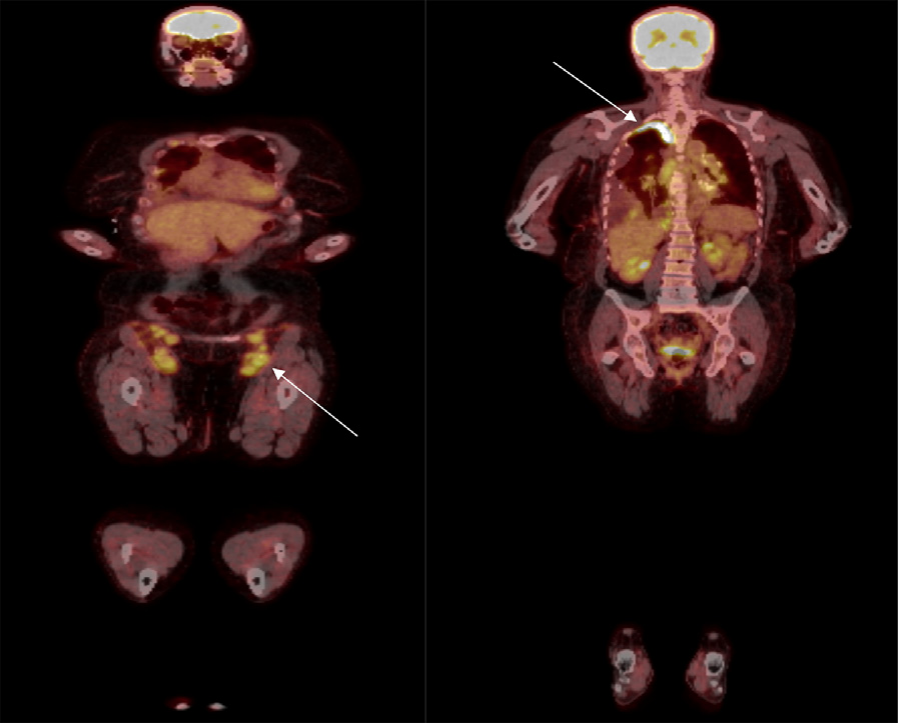
PET-CT coronal windows showing tracer uptake in the inguinal nodes bilaterally (white arrow) suggesting active disease tracer along with uptake in the pleura of the right hemithorax (white arrow) representing reactive granulomatosis related to talc pleurodesis.

**Figure 4:**
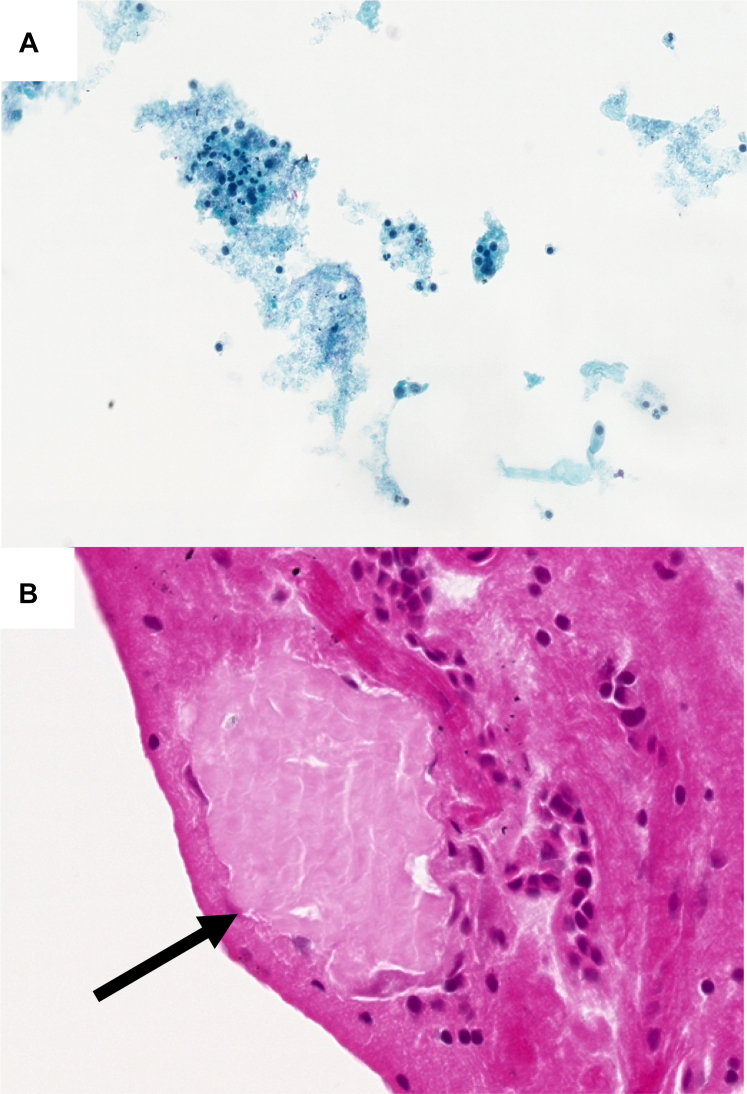
A, Cell block showing scattered lymphocytes and background blood, fibrin, and occasional pigment-laden histiocytes, in keeping with lymph node sampling. B, Cell block from the same lymph node demonstrating pink clustered amorphous material (black arrow) as contrasted from background fibrin with entrapped inflammatory cells. Congo red was positive for amyloid (scale bars 1000um and 50um) .

PET-CT scan demonstrated active disease involving inguinal nodes bilaterally with tracer uptake in enlarged retroperitoneal and axillary nodes and the right hemithorax (Figs 3A, 3B). Bone marrow biopsy was performed demonstrating 10% plasmacytosis with light chain restriction. Endobronchial ultrasound revealed large lower paratracheal lymph nodes, and a biopsy was taken from a right paratracheal lymph node. This contained amorphous eosinophilic material suggestive of amyloid (Figs 4A, 4B). Congo red stain was positive for amyloid deposition.

Multidisciplinary consensus was that the recurrent pleural effusion and lymphadenopathy were due to systemic AL amyloidosis. Initial management of the effusion was with repeated ultrasound-guided chest drain insertion in a regional hospital while awaiting diagnosis. After diagnosis, talc pleurodesis was undertaken, but the effusion recurred requiring the insertion of an IPC. After commencing chemotherapy treatment, the IPC was removed. Since, there was some reaccumulation of the effusion; however, the patient was asymptomatic of the same. After 6 cycles of chemotherapy with rituximab and bendamustine, PET-CT scan revealed disease progression, prompting the need for second-line chemotherapy with ixazomib, revlimid and dexamethasone. The patient tolerated second-line chemotherapy well and at the time of drafting this report was due for a follow-up PET scan in the next 2 months to look for disease progression and treatment efficacy.

## Clinical Pearls

1. *The possibility of amyloidosis should be considered when a refractory pleural effusion or undetermined effusion is encountered, and consideration should be given to thoracoscopic examination.*
2. *Effusions secondary to amyloidosis can be transudates or exudates and can be unilateral or bilateral.*
3. *Definitive identification of the subset of amyloid is crucial to avoid therapeutic errors.*
*Diagnosis of amyloidosis should be based on tissue biopsy and multidisciplinary team discussion. The affected tissue typically shows apple-green birefringence after Congo red staining under polarized light.*
4. *Pleural effusions associated with amyloidosis are often refractory to diuresis and drainage. A combination of systemic chemotherapy along with use of talc pleurodesis or IPC appears to be the best choice of management given current data*.

## Financial/Nonfinancial Disclosures

None declared.
